# Plasma metagenomic sequencing testing for diagnosis of invasive fungal infection in children and young adults

**DOI:** 10.1128/spectrum.00699-26

**Published:** 2026-07-14

**Authors:** Mario M. Landa, Alexa Mendoza, Jenna Rossoff, Ayelet Rosenthal, Sonali Chaudhury, William J. Muller

**Affiliations:** 1 Division of Infectious Diseases, Ann & Robert H. Lurie Children’s Hospital of Chicago, Chicago, Illinois, USA; 2 Stanley Manne Children’s Research Institute, Chicago, Illinois, USA; 3 Division of Pediatric Hematology, Oncology and Stem Cell Transplant, Ann & Robert H. Lurie Children’s Hospital of Chicago, Chicago, Illinois, USA; 4 Department of Pediatrics, Northwestern University Feinberg School of Medicine 12244, Chicago, Illinois, USA; Indiana University School of Medicine, Indianapolis, Indiana, USA

**Keywords:** fungal diagnosis, metagenomic next-generation sequencing, fungal infection

## Abstract

Invasive fungal infection (IFI) is challenging to diagnose, often involving invasive sampling. Plasma cell-free metagenomic next-generation sequencing (mNGS) has shown promise in diagnosing infections, but data are limited on specific clinical scenarios in which this test is most helpful. We conducted a retrospective single-center study of children and young adults with high-risk conditions evaluated for IFI between December 2016 and November 2024. Clinical concern for IFI was indicated by (i) evaluation with both serum β-D-glucan and galactomannan testing, (ii) either or both of CT scans of sinuses and chest, and (iii) antifungal treatment either started or broadened. Episodes in which mNGS testing was sent within 30 days of initiation or broadening of antifungal coverage were evaluated to determine the diagnostic performance of mNGS testing, using EORTC-MSG criteria for proven or probable IFI as the comparator. We identified 227 episodes in 180 high-risk patients consistent with clinical concern for IFI. Of these, 45 episodes met EORTC-MSG criteria for proven/probable IFI. Plasma mNGS testing was sent in 36 episodes and identified the causative organism in 28. Positive and negative percent agreement for diagnosis of proven/probable IFI in this population was 77.8% and 90.4%, respectively. Among proven/probable cases with mNGS testing, *Candida* and *Aspergillus* were the most commonly identified fungi. Plasma mNGS testing in pediatric and young adult patients at risk for IFI compares favorably with diagnostic criteria used for IFI diagnosis and may be added to the diagnostic evaluation of patients at high-risk of IFI.

**IMPORTANCE:**

Performance of plasma mNGS testing for diagnosis of invasive fungal infection in high-risk pediatric and young adult patients was comparable to the combination of fungal culture and targeted PCR from invasively acquired samples, suggesting that it might allow earlier diagnosis for some patients.

## INTRODUCTION

Invasive fungal infection (IFI) is an important cause of morbidity and mortality in immunocompromised patients. Diagnosis often relies on indirect testing, including blood assays for evidence of fungal elements, such as β-D-glucan and *Aspergillus* galactomannan, and suggestive imaging findings (1). Invasive procedures, including bronchoalveolar lavage (BAL) and tissue biopsy, are often pursued to establish a diagnosis. These procedures come with risk in patients who are often clinically fragile, with low platelet counts and possible respiratory insufficiency. Importantly, children often present with different manifestations of IFI than adults (2).

Plasma metagenomic sequencing (mNGS) testing has shown promise in the diagnosis of infections caused by organisms that may be difficult to detect by conventional testing. Data are emerging for specific clinical scenarios in which this test may be most useful, including infective endocarditis (3), bacterial sepsis (4), pulmonary infections after hematopoietic stem cell transplantation (SCT) or in other immunocompromising conditions (5–8), and periprosthetic joint infections (9).

Our large academic pediatric medical center has selectively employed plasma mNGS testing for difficult diagnoses suspected of an infectious cause since late 2016, often for evaluation of IFI in children with immunocompromising conditions. In the current study, we evaluate the performance characteristics of this test in high-risk immunocompromised children and young adults with a clinical concern for IFI.

## MATERIALS AND METHODS

### Study design

We retrospectively evaluated test performance characteristics of plasma mNGS in high-risk pediatric and young adult (age <25 years at the time of evaluation for IFI) patients undergoing evaluation for IFI. Patients were eligible for inclusion in the study if they had an underlying condition conferring elevated risk for IFI (Table 1). Individual episodes indicating clinical concern for IFI were based on test ordering characteristics and initiation or broadening of antifungal coverage by the treating team, according to the criteria in Table 1. We considered the episode start date as the date on which antifungal coverage was either initiated or broadened. Separate episodes for an individual patient needed to include the evaluations listed in Table 1 and to occur at least 30 days apart, with the earlier episode not resulting in a diagnosis of IFI (as defined below) or having a positive mNGS test for a fungus.

**TABLE 1:** Definitions of eligible patients and episodes concerning for IFI

Term	Definition
Immunocompromising diagnoses conferring elevated risk for IFI	Diagnosis of leukemia (acute lymphoblastic leukemia [ALL], acute myeloid leukemia [AML], or myelodysplastic syndrome [MDS]), lymphoma, or other cancer and receiving induction, consolidation, or maintenance immunosuppressive chemotherapy Receipt of an allogeneic stem cell transplant (SCT), chimeric antigen receptor (CAR-T) treatment, or other cell therapy (including donor lymphocyte infusion after SCT) within the prior 180 days (if not being treated for graft-vs-host disease GVHD]) or within the prior year (if being treated for GVHD) Diagnosis of aplastic anemia Diagnosis of other immunocompromising conditions (including genetic immune dysregulation syndromes, hemophagocytic lymphohistiocytosis, and Langerhans cell histiocytosis)
Characteristics of a clinical episode considered concerning for IFI	Patient underwent evaluation with both serum β-D-glucan and serum *Aspergillus* galactomannan testing, AND either or both of computed tomography (CT) scans of sinuses and chest Antifungal treatment was either started or broadened. “Broadened” refers to one or more of (i) increasing dose from a prophylactic dose to treatment dose (for example, increasing micafungin dosing from 1 mg/kg daily to 3 mg/kg or more daily), (ii) adding an additional antifungal agent to provide coverage for more fungal genera (for example, adding liposomal amphotericin B to voriconazole to cover *Mucor* spp.), or (iii) changing from an antifungal, which is considered to provide narrower coverage to one anticipated to have efficacy against additional fungal genera (for example, changing from voriconazole to liposomal amphotericin B for coverage of *Mucor* spp.).^*a*^ All evaluations and antifungal changes needed to occur within a 7-day window for the event to qualify as an episode. The results of these evaluations did not factor into whether any event qualified as an episode (that is, an episode could be qualifying even if serum testing and CT scans were not concerning for IFI).

^*a*^ Our center’s specific practices for fungal prophylaxis in high-risk patients evolved over the course of the study. In general, ALL patients considered at highest risk of fungal infections received fluconazole prophylaxis during their high-risk cycles. Most AML patients received micafungin or voriconazole prophylactically during periods of neutropenia. Early in the study period, stem cell transplant recipients generally received fluconazole prophylaxis, with increasing use of micafungin and voriconazole over time in patients at higher risk. Patients with aplastic anemia or inborn errors of immunity generally received fluconazole as prophylaxis.

Our electronic medical record (EMR) was searched based on ICD-10 codes to generate a list of all patients under age 25 years with immunocompromising diagnoses treated between 20 December 2016 and 1 November 2024. This start date was based on when mNGS testing first became available for clinical diagnostic use at our institution. For each patient, the EMR was queried to identify dates and results for serum β-D-glucan testing, serum *Aspergillus* galactomannan testing, CT scan of sinuses, and CT scan of chest. Results for CT scans of abdomen and/or pelvis were also collected. Charts were individually reviewed to confirm that tests were sent within a 7-day window and whether antifungal coverage was started or broadened within that window, with data compiled into a study database for analysis. Individual episodes starting prior to 1 November 2024 were included.

### Plasma mNGS testing

Whole blood was collected and processed as previously described for analysis at the Karius CLIA/CAP laboratory (Redwood City, CA) (10). The decision to send mNGS testing was based on the clinical team treating the patient; there was not an institutional algorithm, though testing was often considered in patients when there was concern for IFI or other undiagnosed infection. Prior to March 2022, mNGS testing for most episodes was in conjunction with infectious diseases consultation; during and after that month, an infectious diseases provider was required to sign the order for mNGS testing as part of diagnostic stewardship. Blood was analyzed per the Karius laboratory protocol, including extraction of cfDNA from plasma, preparation of NGS libraries, sequencing, and alignment to a curated pathogen database (11). The Karius report has changed over the time period analyzed from an unquantified list of pathogens to current reporting which includes an approximate amount of pathogen as “molecules per milliliter” (MPM). For this study, mNGS tests were interpreted as positive for a fungus (when one was identified), independent of any MPM value. If more than one fungus was identified in a patient by mNGS (or other) testing during an episode, each organism was considered separately in the evaluation for proven or probable IFI. Since *Aspergillus flavus* and *Aspergillus oryzae* were frequently reported together from mNGS testing, they were considered as a single identification for the purposes of analysis.

### IFI episode characterization

In the absence of a diagnostic gold standard test for IFI, we applied criteria from the European Organization for Research and Treatment of Cancer Invasive Fungal Infections Cooperative Group and the National Institute of Allergy and Infectious Diseases Mycoses Study Group (EORTC-MSG) (1, 2) to each episode to classify patients as having proven or probable IFI as “disease-positive” cases. Specifically, patients were considered to have proven IFI if mold or yeast was recovered by microscopic analysis or culture of a normally sterile site, or to have probable IFI if an at-risk host met a clinical criterion (i.e., concerning findings on CT, magnetic resonance imaging [MRI], bronchoscopic, sinus, or ophthalmologic analysis) and a mycologic criterion (i.e., direct detection of mold in nonsterile sites such as sputum, BAL, sinus aspirate, or positive galactomannan or β-D-glucan). In some cases, an individual episode had more than one microbiologic finding satisfying criteria for proven/probable IFI; these were considered as single episodes in the analyses of laboratory test performance. Results of mNGS testing were not used in the categorization of an episode as proven or probable IFI.

Imaging findings in children with IFI may differ from those in adults; accordingly, we considered as concerning findings on CT for lower respiratory tract fungal disease the presence of at least one of the following signs: nodule >5 mm, segmental or lobar consolidation, cavitary lesion, or wedge-shaped lesion/consolidation (12–14). CT imaging findings considered as consistent with fungal sinus infection included partial or complete sinus opacification with or without bony erosion (15). CT findings on abdominal imaging that were considered to be potentially consistent with fungal infection included lucencies or hypodensities in the liver and/or spleen, enlargement of the liver and/or spleen, or bowel wall thickening (14). In all cases, imaging findings needed to be new for the scan associated with the episode to be considered as potential evidence for fungal infection.

Each case was separately reviewed by three of the authors (J. Rossof, A. Rosenthal, and W. J. Muller) to determine whether criteria for proven or probable IFI were met. In episodes for which there was not consensus, re-review and discussion led to a consensus opinion on the classification.

Episodes that did not meet criteria for proven or probable IFI were classified as having no evidence of IFI. For the purposes of our study and consistent with the initial EORTC-MSG criteria (1), *Pneumocystis carinii* pulmonary infections were not categorized as proven or probable IFI.

### Statistical analysis

Mean and median values for patient characteristics were summarized for continuous and categorical values as appropriate, with comparisons using χ^2^ or Fisher’s exact test when appropriate for categorical variables and the Mann-Whitney U test comparisons of numeric data. Test performance characteristics of mNGS testing for proven/probable IFI (“disease-positive”) as defined above were calculated based on identification of a fungus from a test sent within 30 days of the episode date, using a start date corresponding to the date of initiation or broadening of antifungal coverage. Positive percent agreement (PPA) and negative percent agreement (NPA) with EORTC-MSG criteria are reported rather than sensitivity, specificity, or predictive values, given the absence of a true diagnostic gold standard for IFI (16). Predictive values were not calculated, as they require a known true disease status. Confidence intervals used a 95% level, applying the Exact (Clopper–Pearson) method. All analyses were conducted in R (version 4.4.2) (17).

## RESULTS

### Patient characteristics

**Fig 1:**
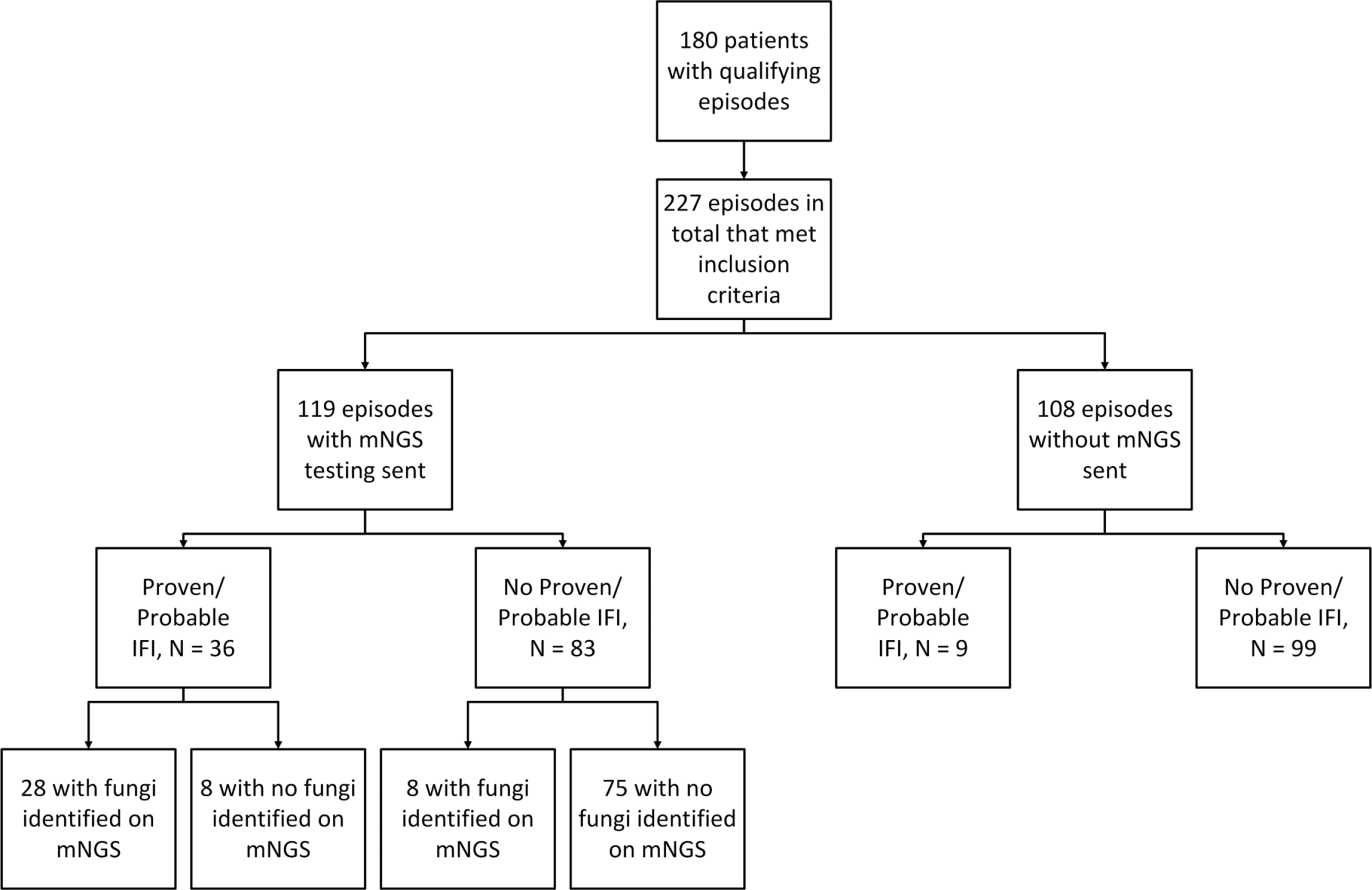
Study flowchart.

A total of 180 patients with an underlying diagnosis conferring risk for IFI experienced 227 episodes that met criteria indicating clinical concern for IFI (Fig. 1). There were no statistical differences in the sex, race, or ethnicity of patients diagnosed with proven/probable IFI compared with patients with eligible episodes that did not result in a diagnosis of proven/probable IFI (Table 2).

**TABLE 2:** Characteristics of patients and episodes included in the study (no proven/probable IFI vs proven/probable IFI)

Patient or episode	Overall	No proven or probable IFI	Proven or probable IFI	*P*-value^*a*^
Patients				
Total	180	141	39	
Sex				
Female	72 (40.0%)	58 (41.1%)	14 (35.9%)	0.555
Male	108 (60.0%)	83 (58.9%)	25 (64.1%)	
Race				
Asian	8 (4.4%)	6 (4.3%)	2 (5.1%)	0.135
Black or African American	17 (9.4%)	16 (11.3%)	1 (2.6%)	
Multiple or other	82 (45.6%)	67 (47.5%)	15 (38.5%)	
White	73 (40.6%)	52 (36.9%)	21 (53.8%)	
Ethnicity				
Hispanic or Latine	74 (41.1%)	59 (41.8%)	15 (38.5%)	0.823
Not Hispanic or Latine	104 (57.8%)	80 (56.7%)	24 (61.5%)	
Declined or unknown	2 (1.1%)	2 (1.4%)	0 (0.0%)	
Episodes				
Total	227	182 (80.2%)	45 (19.8%)	
Median age at time of episode, years [IQR]	10.1 [4.4, 15.3]	10.1 [4.2, 15.4]	10.1 [5.8, 14.5]	0.374
Underlying diagnosis at time of episode^*b*^				
ALL	87 (38.3%)	60 (33.0%)	27 (60.0%)	
AML, JMML, MDS	36 (15.9%)	27 (14.8%)	9 (20.0%)	
Lymphoma	13 (5.7%)	10 (5.5%)	3 (6.7%)	
Other cancers receiving immunosuppressive medications	13 (5.7%)	13 (7.1%)	0 (0%)	<0.001
Aplastic anemia/Evan’s syndrome	10 (4.4%)	8 (4.4%)	2 (4.4%)	
STAT3 gain-of-function immune dysregulation, Langerhans cell histiocytosis, HLH	4 (1.8%)	3 (1.6%)	1 (2.2%)	
Allogeneic SCT, CAR-T treatment, or other cell therapy	64 (28.2%)	61 (33.5%)	3 (6.7%)	
Positive findings on clinical IFI evaluation				
Serum β-D-glucan ≥80 pg/mL	47 (20.7%)	27 (14.8%)	20 (44.4%)	<0.001
Serum *Aspergillus* galactomannan >0.5	16 (7.0%)	4 (2.2%)	12 (26.7%)	<0.001
≥1 abnormality on imaging (CT chest/sinus or abdomen/pelvis)	157 (69.2%)	123 (67.6%)	34 (75.6%)	0.300
≥1 abnormality on imaging (CT chest/sinus only)	132 (58.1%)	101 (55.5%)	31 (68.9%)	0.103
Positive culture for fungus^*c*^	30 (13.2%)	2 (1.1%)	28 (62.2%)	<0.001
mNGS testing sent	119 (52.4%)	83 (45.6%)	36 (80.0%)	<0.001
Fungus identified on mNGS testing	36 (15.9%)	8 (4.4%)	28 (62.2%)	<0.001
Procedures performed				
Bronchoalveolar lavage	30 (13.2%)	25 (13.7%)	5 (11.1%)	0.641
Lung biopsy	17 (7.5%)	7 (3.8%)	10 (22.2%)	<0.001
Liver biopsy	9 (4.0%)	5 (2.7%)	4 (8.9%)	0.079
Sinus sampling	11 (4.8%)	2 (1.1%)	9 (20.0%)	<0.001

^*a*^ Statistical comparison is between patients with an eligible episode that ultimately were not diagnosed with proven or probable IFI and those who ultimately were classified as having proven or probable IFI (top part of table), or between eligible episodes that ultimately resulting in a proven or probable IFI diagnosis compared with those that did not (bottom part of Table).

^*b*^ ALL, acute lymphoblastic leukemia; AML, acute myeloid leukemia; JMML, juvenile myelomonocytic leukemia; MDS, myelodysplastic syndrome; HLH, hemophagocytic lymphohistiocytosis; SCT, stem cell transplantation; the Freeman-Halton extension of Fisher’s exact test using a Monte Carlo approximation with 10,000 replicates was applied to assess differences in the distribution of underlying diagnoses.

^*c*^ Cultures from non-sterile sites were excluded, including sputum and endotracheal tube cultures. The two positive cultures in patients without proven/probable IFI represented episodes involving *Candida albicans* that did not meet criteria for proven/probable IFI, though they were considered as disease-causing. One was collected from a superficial skin abscess at the site of a port, and the other was from an infected pancreatic pseudocyst in a patient with pancreatitis.

There were 45 episodes meeting criteria for proven/probable IFI (19.8% of all episodes, Table 2). The proportion of patients diagnosed with proven/probable IFI with ALL as their underlying disease was significantly higher than their representation in the overall population of patients experiencing episodes, while the proportion who had received cellular therapy (mostly SCT) was significantly lower. Patients with qualifying episodes were not more likely to have mNGS testing sent based on demographic factors or underlying diagnoses (Table S1).

Clinical evaluation of patients for IFI included serum β-D-glucan, galactomannan, and imaging. As anticipated, episodes that resulted in diagnoses of proven/probable IFI were more likely to have abnormal findings from these tests when compared with those episodes not eventually resulting in proven/probable IFI (Table 2). Invasive sampling of the lung or sinuses was also more likely to occur in episodes of proven/probable IFI. mNGS testing was more likely to be sent during episodes which had abnormal findings from routine IFI evaluations and in which invasive procedures were pursued (Table S1).

### Fungal testing results in episodes of proven/probable IFI

Plasma mNGS testing was sent within 30 days of a start or broadening of antifungal coverage in 36 (80%) of the episodes that met criteria for proven/probable IFI. In 27 of these episodes (75%), a causative fungus was identified by routine microbiologic testing, including culture and fungal PCR (Table S2). Of these 27 episodes, mNGS testing identified the same organism in 19 cases (70%; further information on discrepant test results is provided later). Of nine proven/probable episodes in which standard microbiologic testing did not identify a causative organism, mNGS testing identified a plausible causative organism in every case. For six of these cases, standard microbiologic testing, including morphologic appearance of the organism on pathologic analysis, and indirect testing with elevated serum galactomannan were consistent with *Aspergillus* spp. as identified by mNGS testing (Table S2).

Among proven/probable cases with mNGS testing, *Candida* and *Aspergillus* were the most commonly identified genera, with *Aspergillus* in 13 episodes and *Candida* in 9. In five episodes, more than one fungal genus was identified by either or both of standard microbiologic testing or mNGS. In four of these episodes, mNGS identified polymicrobial infection which was supported by clinical presentation and standard microbiologic testing data, in which *Aspergillus* infection was consistent with pathologic analysis or culture of biopsy samples and by indirect testing, and other organisms (*Candida* or *Rhizopus*) by culture data. For one episode of invasive fungal sinusitis (episode 117), two mNGS tests were sent, one of which identified *Alternaria* and the other *Rhizopus*; the clinical impression was that *Alternaria* was likely present but perhaps a superficial infection. In another episode of invasive fungal sinusitis (episode 133), pathologic evaluation suggested two organisms, one of which was supported by culture of *Exserohilum rostratum* and the other by mNGS identification of *Aspergillus* spp. from consecutive tests.

### mNGS test performance for proven/probable IFI

In addition to the 36 episodes of proven/probable IFI in which mNGS testing was sent, there were 83 episodes involving 74 patients in which plasma mNGS was sent but proven/probable IFI was not diagnosed. A fungus was identified in eight of these tests. Based on these data, positive and negative percent agreement (95% CI) of plasma mNGS for diagnosis of proven/probable IFI in this population was 77.8% (60.9, 89.9) and 90.4% (81.9, 95.8), respectively (Table 3). Positive percent agreement of mNGS testing was comparable to the overall analysis when applied only to episodes of invasive aspergillosis, or in patients in whom testing was sent within 5 days of the episode start, with abnormalities on imaging, or with neutropenia at the time of the episode. In comparison, the test performance characteristics of routine clinical evaluations, including serum β-D-glucan and *Aspergillus* galactomannan, had much lower positive percent agreement, while abnormalities on chest or sinus CT imaging had lower positive and negative percent agreement for diagnosis of proven/probable IFI (Table 4). Positive fungal culture or PCR from an episode was associated with comparable test performance characteristics to mNGS testing (Table 4).

**TABLE 3:** Performance characteristics of plasma mNGS testing for diagnosis of proven/probable IFI

Plasma mNGS testing	All qualifying episodes (*N* = 119)	Invasive *Aspergillus* cases (*N* = 100)^*a*^	mNGS testing sent within 5 days of antifungal change^*b*^ (*N* = 90)	Among patients with abnormal chest/sinus CT^*c*^ (*N* = 94)	Among patients with neutropenia^*d*^ (*N* = 75)
Positive percent agreement (95% CI)	77.8 (60.9, 89.9)	76.5 (50.1, 93.2)	81.5 (61.9, 93.7)	86.2 (68.3, 96.1)	80.0 (61.4, 92.3)
Negative percent agreement (95% CI)	90.4 (81.9, 95.8)	98.8 (93.5, 100)	92.1 (82.4, 97.4)	89.2 (79.1, 95.6)	86.7 (73.2, 95.0)

^*a*^ Analysis of test performance characteristics for *Aspergillus* episodes included proven/probable episodes involving *Aspergillus* and excluded those caused by other fungi.

^*b*^ Episode start date defined as the date on which antifungal coverage was either initiated or broadened (see Materials and Methods).

^*c*^ Abnormal imaging (defined in the Materials and Methods section) included new findings on pulmonary CT showing nodule(s) >5 mm, segmental or lobar consolidation, cavitary lesion, or wedge-shaped lesion/consolidation; on sinus CT of partial or complete sinus opacification with or without bony erosion; or on abdominal CT of lucencies or hypodensities in the liver and/or spleen, enlargement of the liver and/or spleen, or bowel wall thickening.

^*d*^ Neutropenia defined as an absolute neutrophil count of <500 cells/mm^3^ for ≥10 days, temporally related to the start date of the episode.

**TABLE 4:** Performance characteristics of routine clinical evaluations for diagnosis of proven/probable IFI

Routine clinical testing^*a*^	Serum β-D-glucan (*N* = 227)	Serum *Aspergillus* galactomannan^*b*^ (*N* = 227)	Abnormal chest/sinus CT^*c*^ (*N* = 227)	Positive fungal culture or PCR^*d*^ (*N* = 143)
Positive percent agreement (95% CI)	44.4 (29.6, 60.0)	47.8 (26.8, 69.4)	68.9 (53.4, 81.8)	83.3 (68.6, 93.0)
Negative percent agreement (95% CI)	85.2 (79.2, 90.0)	97.8 (94.5, 99.4)	44.5 (37.2, 52.0)	93.1 (86.2, 97.2)

^*a*^ Note that these tests may be included in the EORTC-MSG criteria used to define proven/probable IFI for any given episode: the calculation of positive percent agreement and negative percent agreement in this section of the Table is for these tests in isolation compared to the eventual classification of the episode.

^*b*^ Analysis of test performance characteristics for *Aspergillus* episodes included proven/probable episodes involving *Aspergillus* and excluded those caused by other fungi.

^*c*^ Abnormal imaging (defined in the Materials and Methods section) included new findings on pulmonary CT showing nodule(s) >5 mm, segmental or lobar consolidation, cavitary lesion, or wedge-shaped lesion/consolidation; on sinus CT of partial or complete sinus opacification with or without bony erosion; or on abdominal CT of lucencies or hypodensities in the liver and/or spleen, enlargement of the liver and/or spleen, or bowel wall thickening.

^*d*^ Includes episodes in which tissue samples (BAL or biopsy) were evaluated by fungal culture or targeted PCR for fungi.

### Additional analysis of false positive and negative mNGS testing

There were eight episodes in which mNGS testing identified a fungus in patients without proven/probable IFI (false positives; Table S3). In five of these episodes, an organism identified by mNGS testing was a plausible cause of infection based on results of standard microbiologic testing and clinical impression. One case in which mNGS identified *Pneumocystis jirovecii* was clinically consistent with this infection, with *Candida albicans* identified on the same test considered a false positive.

In eight episodes of proven/probable IFI, mNGS testing failed to identify the causative organism (false negatives; Tables S2 and S4). Five of these tests failed to identify any microbes. In one case, mNGS testing identified *Pneumocystis jirovecii* pneumonitis, which was consistent with the patient’s clinical presentation, though *Aspergillus fumigatus* grew from a lung biopsy sample late in the incubation period. In one other case, *Streptococcus agalactiae* was identified on mNGS testing in a patient with concurrent disseminated infection with that organism.

## DISCUSSION

Diagnosis of IFI in high-risk pediatric patients is challenging. Plasma mNGS testing in our study had high positive and negative percent agreement with EORTC-MSG defined proven/probable IFI in high-risk pediatric patients, with test performance characteristics comparable to the combination of fungal culture and targeted PCR testing of samples acquired by invasive sampling (BAL or biopsy). Our positive and negative percent agreement of fungal testing from BAL or biopsy samples in children were also comparable to prior reports, where galactomannan and/or fungal PCR testing of BAL has been found to have positive and negative percent agreement of 82%–96% for diagnosis of pulmonary aspergillosis (18–20). Similar performance has been reported for diagnosis of IFI using fungal PCR from tissue samples, though culture has lower positive percent agreement (21, 22). Our observation that mNGS testing may have similar diagnostic performance as invasive sampling, combined with those made by prior authors (6), suggests that in some patients it may allow for earlier diagnosis, possibly allowing these procedures may be avoided in some cases.

Prior studies of mNGS testing for IFI diagnosis focused on adult patients have also suggested good diagnostic performance. A study of stored plasma samples collected from 114 adult SCT recipients with pneumonia within 14 days of diagnosis of IFI reported overall positive and negative percent agreement of mNGS for proven/probable IFI of 51% and 82%, respectively (5). Recent studies of prospectively collected plasma samples from immunocompromised adult patients suspected of pulmonary IFI found plasma mNGS had positive and negative percent agreement of 44%–48% and 97%–100%, respectively, for diagnosis of proven/probable IFI (6, 23). We found generally higher positive percent agreement than was reported from these studies. Additionally, these studies in adults found that positive percent agreement of mNGS testing was higher in non-Aspergillus IFI, which we did not observe (Table 3).

Establishing a microbiologic diagnosis is an important goal of evaluation for IFI. Although the negative percent agreement for mNGS testing in our study was high, there were several false negatives. In five of the eight cases in which mNGS testing did not identify the causative organism in a patient with proven/probable IFI (Table S4), no organisms were identified, and diagnosis was established through culture or PCR testing. In three cases in which mNGS testing was positive, the organism identified was a plausible contributor to the patient’s presentation. Episode 72 involved a clinical presentation consistent with *Pneumocystis jirovecii* pneumonia, for which the patient was treated; the positive culture from a lung biopsy sample obtained several days after presentation grew *Aspergillus* late in the incubation period.

Conversely, falsely identifying an organism in a high-risk patient can lead to overtreatment, often with nephrotoxic or hepatotoxic medications in patients already receiving multiple medications. However, of the eight episodes that did not meet criteria for proven/probable IFI but in which mNGS testing identified a fungus, five involved organisms plausibly contributing to disease in the patient (Table S4). One of the remaining cases involved an organism (*Aureobasidium pullulans*) that is rarely pathogenic and was not considered to be clinically relevant, and two involved detection of *Candida* species in patients without other clinical findings consistent with that infection. Taken together, review of the episodes with mNGS testing considered falsely positive or falsely negative in combination with laboratory and imaging findings in these patients support potential additional value of mNGS testing in establishing a contributing microbiologic cause in some cases, independent of whether they fully meet criteria for proven/probable IFI. This is consistent with observations made from a prior prospective observational study of 173 adult patients at risk for IFI undergoing bronchoscopy for presumed pneumonia, which found that mNGS testing between −1 and +5 days from BAL identified a microbiologic cause in 12.1% of those in whom usual care testing was negative and in 23.1% of those in whom non-invasive usual care testing was negative (7).

We found lower positive percent agreement of blood tests that assess for fungal elements (β-D-glucan or galactomannan) in the diagnosis of proven/probable disease than mNGS or other testing (Table 4), consistent with prior studies showing variable performance that is generally lower than other methods for IFI diagnosis (24). Addition of blood PCR for fungal DNA to these tests may increase diagnostic value (25); however, a role for combinations of blood tests (potentially including plasma mNGS testing) in diagnosing IFI to decrease the need for invasive procedures remains to be studied further.

Our study has certain strengths, including the use of clinically defined criteria for identifying our study population, intended to avoid bias in determining the test performance. We focused on high-risk patients for whom there was objective evidence of clinical concern for IFI based on the actions of the clinical treating team. Our center’s use of mNGS testing over about a 7-year period provided a sufficient number of episodes in which mNGS testing was done to adequately assess test performance. Our finding that approximately 20% of all episodes meeting our inclusion criteria led to a diagnosis of proven/probable IFI is comparable to literature reports in similar populations, which have found proven/probable IFI in roughly 15%–20% (26–28).

There are limitations to our study. As a single-center, retrospective study, the data may not be completely generalizable to other clinical settings. Additionally, relative to the number of proven/probable diagnoses made in the different populations, mNGS testing was sent more often on SCT patients relative to other underlying high-risk conditions, particularly ALL; 60% of the total number of proven/probable infections were diagnosed in ALL patients, while only 39% of the mNGS tests were sent from that cohort, compared with 6.7% and 24%, respectively, in SCT patients (comparing Table 1 with Table S1). Whether this could be due to differences in the threshold for test ordering between the different treating teams, differences in the threshold for preferring invasive tests to mNGS testing, or other factors is unclear. Finally, the laboratory conducting mNGS testing for our center changed its analysis and reporting practices over the period analyzed, which may have unclear effects on the results reported.

### Conclusions

Plasma mNGS testing in pediatric patients with underlying diseases conferring risk for IFI has favorable performance characteristics, comparable to those seen with culture or microbiologic testing of invasively obtained samples. This test could be added to the evaluation of high-risk patients with clinical episodes concerning for IFI.

## Data Availability

The data underlying this article will be shared on reasonable request to the corresponding author.

## SUPPLEMENTAL MATERIAL

Supplemental Material spectrum.00699-26-s0001.docx Additional data tables.

ASM does not own the copyrights to Supplemental Material that may be linked to, or accessed through, an article. The authors have granted ASM a non-exclusive, world-wide license to publish the Supplemental Material files. Please contact the corresponding author directly for reuse.

## References

[1] De Pauw B, Walsh TJ, Donnelly JP, Stevens DA, Edwards JE, Calandra T, Pappas PG, Maertens J, Lortholary O, Kauffman CA, et al.. 2008. Revised definitions of invasive fungal disease from the European Organization for Research and Treatment of Cancer/Invasive Fungal Infections Cooperative Group and the National Institute of Allergy and Infectious Diseases Mycoses Study Group (EORTC/MSG) consensus group. Clin Infect Dis 46:1813–1821. doi:10.1086/588660 18462102 PMC2671227

[2] Donnelly JP, Chen SC, Kauffman CA, Steinbach WJ, Baddley JW, Verweij PE, Clancy CJ, Wingard JR, Lockhart SR, Groll AH, et al.. 2020. Revision and update of the consensus definitions of invasive fungal disease from the European organization for research and treatment of cancer and the mycoses study group education and research consortium. Clin Infect Dis 71:1367–1376. doi:10.1093/cid/ciz1008 31802125 PMC7486838

[3] Eichenberger EM, Degner N, Scott ER, Ruffin F, Franzone J, Sharma-Kuinkel B, Shah P, Hong D, Dalai SC, Blair L, Hollemon D, Chang E, Ho C, Wanda L, de Vries CR, Fowler VG, Ahmed AA. 2023. Microbial cell-free DNA identifies the causative pathogen in infective endocarditis and remains detectable longer than conventional blood culture in patients with prior antibiotic therapy. Clin Infect Dis 76:e1492–e1500. doi:10.1093/cid/ciac426 35684984 PMC10169441

[4] Brenner T, Decker SO, Vainshtein Y, Grumaz S, Manoochehri M, Feißt M, Seidel-Glätzer A, Pletz MW, Bracht H, Berger MM, et al.. 2025. Improved pathogen identification in sepsis or septic shock by clinical metagenomic sequencing. J Infect 91:106565. doi:10.1016/j.jinf.2025.106565 40774415

[5] Hill JA, Dalai SC, Hong DK, Ahmed AA, Ho C, Hollemon D, Blair L, Maalouf J, Keane-Candib J, Stevens-Ayers T, Boeckh M, Blauwkamp TA, Fisher CE. 2021. Liquid biopsy for invasive mold infections in hematopoietic cell transplant recipients with pneumonia through next-generation sequencing of microbial cell-free DNA in plasma. Clin Infect Dis 73:e3876–e3883. doi:10.1093/cid/ciaa1639 33119063 PMC8664431

[6] Sim BZ, Mah JK, Heldman MR, Stanly KL, Hanson KE, Caliendo AM, Andes D, Ostrosky-Zeichner L, Wingard JR, Alexander BD. 2025. Plasma microbial cell-free DNA metagenomic sequencing for diagnosis of invasive fungal diseases among high-risk outpatient and inpatient immunocompromised hosts. Clin Infect Dis 81:1008–1014. doi:10.1093/cid/ciaf170 40176261 PMC12728277

[7] Bergin SP, Chemaly RF, Dadwal SS, Hill JA, Lee YJ, Haidar G, Luk A, Drelick A, Chin-Hong PV, Benamu E, et al.. 2024. Plasma microbial cell-free DNA sequencing in immunocompromised patients with pneumonia: a prospective observational study. Clin Infect Dis 78:775–784. doi:10.1093/cid/ciad599 37815489 PMC10954333

[8] Madut DB, Chemaly RF, Dadwal SS, Hill JA, Lee YJ, Haidar G, Luk A, Drelick A, Chin-Hong PV, Benamu E, et al.. 2024. Clinical utility of plasma microbial cell-free DNA sequencing among immunocompromised patients with pneumonia. Open Forum Infect Dis 11:ofae425. doi:10.1093/ofid/ofae425 39091643 PMC11292041

[9] Echeverria AP, Cohn IS, Danko DC, Shanaj S, Blair L, Hollemon D, Carli AV, Sculco PK, Ho C, Meshulam-Simon G, et al.. 2021. Sequencing of circulating microbial cell-free DNA can identify pathogens in periprosthetic joint infections. J Bone Joint Surg Am 103:1705–1712. doi:10.2106/JBJS.20.02229 34293751

[10] Armstrong AE, Rossoff J, Hollemon D, Hong DK, Muller WJ, Chaudhury S. 2019. Cell‐free DNA next‐generation sequencing successfully detects infectious pathogens in pediatric oncology and hematopoietic stem cell transplant patients at risk for invasive fungal disease. Pediatr Blood Cancer 66:e27734. doi:10.1002/pbc.27734 30941906

[11] Blauwkamp TA, Thair S, Rosen MJ, Blair L, Lindner MS, Vilfan ID, Kawli T, Christians FC, Venkatasubrahmanyam S, Wall GD, Cheung A, Rogers ZN, Meshulam-Simon G, Huijse L, Balakrishnan S, Quinn JV, Hollemon D, Hong DK, Vaughn ML, Kertesz M, Bercovici S, Wilber JC, Yang S. 2019. Analytical and clinical validation of a microbial cell-free DNA sequencing test for infectious disease. Nat Microbiol 4:663–674. doi:10.1038/s41564-018-0349-6 30742071

[12] Burgos A, Zaoutis TE, Dvorak CC, Hoffman JA, Knapp KM, Nania JJ, Prasad P, Steinbach WJ. 2008. Pediatric invasive aspergillosis: a multicenter retrospective analysis of 139 contemporary cases. Pediatrics 121:e1286–94. doi:10.1542/peds.2007-2117 18450871

[13] Ankrah AO, Sathekge MM, Dierckx RAJO, Glaudemans AWJM. 2016. Imaging fungal infections in children. Clin Transl Imaging 4:57–72. doi:10.1007/s40336-015-0159-2 26913275 PMC4752574

[14] Varotto A, Orsatti G, Crimì F, Cecchin D, Toffolutti T, Zucchetta P, Stramare R. 2019. Radiological assessment of paediatric fungal infections: a pictorial review with focus on PET/MRI. In Vivo 33:1727–1735. doi:10.21873/invivo.11663 31662497 PMC6899128

[15] DelGaudio JM, Swain RE, Kingdom TT, Muller S, Hudgins PA. 2003. Computed tomographic findings in patients with invasive fungal sinusitis. Arch Otolaryngol Head Neck Surg 129:236–240. doi:10.1001/archotol.129.2.236 12578456

[16] U.S. Food and Drug Administration. 2007. Guidance for Industry and FDA staff: statistical guidance on reporting results from studies evaluating diagnostic tests. U.S. Food and Drug Administration, Silver Spring, MD. Available from: https://www.fda.gov/downloads/medicaldevices/deviceregulationandguidance/guidancedocuments/ucm071287.pdf

[17] R: a language and environment for statistical computing. R foundation for statistical computing. 2024. Available from: https://www.R-project.org. Retrieved 17 Nov 2025.

[18] de Mol M, de Jongste JC, van Westreenen M, Merkus PJFM, de Vries AHC, Hop WCJ, Warris A, Janssens HM. 2013. Diagnosis of invasive pulmonary aspergillosis in children with bronchoalveolar lavage galactomannan. Pediatr Pulmonol 48:789–796. doi:10.1002/ppul.22670 22949309

[19] D’Haese J, Theunissen K, Vermeulen E, Schoemans H, De Vlieger G, Lammertijn L, Meersseman P, Meersseman W, Lagrou K, Maertens J. 2012. Detection of galactomannan in bronchoalveolar lavage fluid samples of patients at risk for invasive pulmonary aspergillosis: analytical and clinical validity. J Clin Microbiol 50:1258–1263. doi:10.1128/JCM.06423-11 22301025 PMC3318563

[20] Avni T, Levy I, Sprecher H, Yahav D, Leibovici L, Paul M. 2012. Diagnostic accuracy of PCR alone compared to galactomannan in bronchoalveolar lavage fluid for diagnosis of invasive pulmonary aspergillosis: a systematic review. J Clin Microbiol 50:3652–3658. doi:10.1128/JCM.00942-12 22952268 PMC3486225

[21] Buitrago MJ, Aguado JM, Ballen A, Bernal-Martinez L, Prieto M, Garcia-Reyne A, Garcia-Rodriguez J, Rodriguez-Tudela JL, Cuenca-Estrella M. 2013. Efficacy of DNA amplification in tissue biopsy samples to improve the detection of invasive fungal disease. Clin Microbiol Infect 19:E271–7. doi:10.1111/1469-0691.12110 23464751

[22] Gomez CA, Budvytiene I, Zemek AJ, Banaei N. 2017. Performance of targeted fungal sequencing for culture-independent diagnosis of invasive fungal disease. Clin Infect Dis 65:2035–2041. doi:10.1093/cid/cix728 29020284

[23] Huygens S, Schauwvlieghe A, Wlazlo N, Moors I, Boelens J, Reynders M, Chong G-L, Klaassen CHW, Rijnders BJA. 2024. Diagnostic value of microbial cell-free dna sequencing for suspected invasive fungal infections: a retrospective multicenter cohort study. Open Forum Infect Dis 11:ofae252. doi:10.1093/ofid/ofae252 38868302 PMC11166502

[24] Singh S, Singh M, Verma N, Sharma M, Pradhan P, Chauhan A, Jaiswal N, Chakrabarti A, Singh M. 2021. Comparative accuracy of 1,3 beta-D glucan and galactomannan for diagnosis of invasive fungal infections in pediatric patients: a systematic review with meta-analysis. Med Mycol 59:139–148. doi:10.1093/mmy/myaa038 32448907

[25] Gupta P, Ahmad A, Khare V, Kumar A, Banerjee G, Verma N, Singh M. 2017. Comparative evaluation of pan-fungal real-time PCR, galactomannan and (1-3)-β-D-glucan assay for invasive fungal infection in paediatric cancer patients. Mycoses 60:234–240. doi:10.1111/myc.12584 27862370

[26] Sahbudak Bal Z, Yilmaz Karapinar D, Karadas N, Sen S, Onder Sivis Z, Akinci AB, Balkan C, Kavakli K, Vardar F, Aydinok Y. 2015. Proven and probable invasive fungal infections in children with acute lymphoblastic leukaemia: results from an university hospital, 2005-2013. Mycoses 58:225–232. doi:10.1111/myc.12303 25728069

[27] Lehrnbecher T, Schöning S, Poyer F, Georg J, Becker A, Gordon K, Attarbaschi A, Groll AH. 2019. Incidence and outcome of invasive fungal diseases in children with hematological malignancies and/or allogeneic hematopoietic stem cell transplantation: results of a prospective multicenter study. Front Microbiol 10:681. doi:10.3389/fmicb.2019.00681 31040830 PMC6476895

[28] Cesaro S, Tridello G, Castagnola E, Calore E, Carraro F, Mariotti I, Colombini A, Perruccio K, Decembrino N, Russo G, Maximova N, Baretta V, Caselli D. 2017. Retrospective study on the incidence and outcome of proven and probable invasive fungal infections in high-risk pediatric onco-hematological patients. Eur J Haematol 99:240–248. doi:10.1111/ejh.12910 28556426

