# Supplementary material for: Plasma metagenomic sequencing testing for diagnosis of invasive fungal infection in children and young adults
Source: Microbiol Spectr. 2026 Jul 14;14(8):e00699-26. doi: 10.1128/spectrum.00699-26 (PMC13436007; doi:10.1128/spectrum.00699-26)
Supplement: Supplementary file 1 [file spectrum.00699-26-s0001.docx]

**Supplementary Table S1.** Characteristics of patients and episodes included in the study (no mNGS testing sent vs. mNGS testing sent)

| **Patient Specific Variables** | **Overall** | **No mNGS testing sent** | **mNGS testing sent** | **P-value** |
| --- | --- | --- | --- | --- |
| **Total** | 180 | 90 | 90 |  |
| **Sex** |  |  |  |  |
| Female | 72 (40.0%) | 34 (37.8%) | 38 (42.2%) | 0.543 |
| Male | 108 (60.0%) | 56 (62.2%) | 52 (57.8%) |  |
| **Race** |  |  |  |  |
| Asian | 8 (4.4%) | 4 (4.4%) | 4 (4.4%) | 0.154 |
| Black or African American | 17 (9.4%) | 10 (11.1%) | 7 (7.8%) |  |
| Multiple or Other | 82 (45.6%) | 47 (52.2%) | 35 (38.9%) |  |
| White | 73 (40.6%) | 29 (32.2%) | 44 (48.9%) |  |
| **Ethnicity** |  |  |  |  |
| Hispanic or Latine | 74 (41.1%) | 39 (43.3%) | 35 (38.9%) | 0.357 |
| Not Hispanic or Latine | 104 (57.8%) | 49 (54.4%) | 55 (61.1%) |  |
| Declined | 2 (1.1%) | 2 (2.2%) | 0 (0.0%) |  |
| **Episode Specific Variables** |  |  |  |  |
| **Total** | 227 | 108 | 119 |  |
| **Median age at time of episode, yrs [IQR]** | 10.1 [4.4, 15.3] | 10.3 [4.0, 16.3] | 9.9 [4.4, 14.4] | 0.761 |
| **Underlying diagnosis at time of episode**^1^ |  |  |  |  |
| ALL | 87 (38.3%) | 41 (38.0%) | 46 (38.7%) | 0.315 |
| AML, JMML, MDS | 36 (15.9%) | 14 (13.0%) | 22 (18.5%) |  |
| Lymphoma | 13 (5.7%) | 5 (4.6%) | 8 (6.7%) |  |
| Other cancers receiving immunosuppressive medications | 13 (5.7%) | 7 (6.5%) | 6 (5.0%) |  |
| Aplastic anemia/Evan’s syndrome | 10 (4.4%) | 5 (4.6%) | 5 (4.2%) |  |
| STAT3 gain-of-function immune dysregulation, Langerhans cell histiocytosis, HLH | 4 (1.8%) | 0 (0.0%) | 4 (3.4%) |  |
| Allogeneic SCT, CAR-T treatment, or other cell therapy | 64 (28.2%) | 36 (33.3%) | 28 (23.5%) |  |
| **Positive findings on clinical IFI evaluation** |  |  |  |  |
| Serum β-D-glucan ≥80 pg/mL | 47 (20.7%) | 17 (15.7%) | 30 (25.2%) | 0.079 |
| Serum *Aspergillus* galactomannan >0.5 | 16 (7.0%) | 4 (3.7%) | 12 (10.1%) | 0.061 |
| ≥1 abnormality on imaging (CT chest/sinus or abdomen/pelvis) | 157 (69.2%) | 63 (58.3%) | 94 (79.0%) | <0.001 |
| ≥1 abnormality on imaging (CT chest/sinus only) | 132 (58.1%) | 49 (45.4%) | 83 (69.7%) | <0.001 |
| Positive culture for fungus | 30 (13.2%) | 8 (7.4%) | 22 (18.5%) | 0.284 |
| **Procedures performed** |  |  |  |  |
| Bronchoalveolar lavage | 30 (13.2%) | 7 (6.5%) | 23 (19.3%) | 0.004 |
| Lung biopsy | 17 (7.5%) | 4 (3.7%) | 13 (10.9%) | 0.039 |
| Liver biopsy | 9 (4.0%) | 3 (2.8%) | 6 (5.0%) | 0.504 |
| Sinus sampling | 11 (4.8%) | 0 (0.0%) | 11 (9.2%) | 0.001 |

^1^ALL- acute lymphoblastic leukemia; AML- acute myeloid leukemia; JMML- juvenile myelomonocytic leukemia; MDS- myelodysplastic syndrome; HLH- hemophagocytic lymphohistiocytosis; SCT- stem cell transplantation

**Supplementary Table S2.** Results of IFI evaluation including mNGS testing in episodes of proven/probable IFI

| **Episode #** | **Proven/Probable** | **Anatomic location(s)** | **EORTC criteria** | **mNGS result** |
| --- | --- | --- | --- | --- |
| 18 | Probable | Endocarditis | Host criteria: Recent neutropenia^1^  Clinical criteria: Chest CT nodule >5 mm  Mycological criteria:  Serum GM 0.878  β-D-glucan >500 pg/mL | ***Aspergillus oryzae***  ***Aspergillus flavus*** |
| 36 | Probable | Pulmonary | Host criteria: Recent neutropenia  Clinical criteria: Chest CT nodule >5 mm  Mycological criteria:  Serum GM 3.183 | *Escherichia coli*  ***Aspergillus fumigatus***  *Staphylococcus epidermidis* |
| 38 | Proven | Disseminated | Blood culture positive for *Candida tropicalis* | ***Candida tropicalis*** |
| 40 | Proven | Sinus | Pathology with hyphae and angioinvasion | ***Lichthemia corymbifera***  *Staphylococcus epidermidis* |
| 46 | Proven | Sinus | Pathology with hyphae and angioinvasion  Culture positive for *Rhizomucor miehei* (species identified by PCR) | ***Rhizomucor miehei*** |
| 57 | Proven | Disseminated | Blood culture positive for *Candida krusei* | ***Candida krusei***  *Acinetobacter hemolyticus*  Adenovirus C  Cytomegalovirus |
| 62 | Proven | Disseminated | Blood culture positive for *Candida albicans* | ***Candida albicans*** |
| 69 | Probable | Central nervous system | Host criteria: Recipient of an allogeneic stem cell transplant  Clinical criteria:  Focal CNS lesions on MRI  Mycological criteria:  Serum GM >3.750  CSF GM 9.15  β-D-glucan >500 pg/mL | *Enterococcus faecalis*  ***Aspergillus fumigatus***  *Corynebacterium striatum* |
| 72 | Proven^2^ | Pulmonary | Lung biopsy culture positive for *Aspergillus fumigatus* | *Pneumocystis jirovecii*  Torque tenovirus |
| 84 | Probable^3^ | Disseminated | Host criteria: Recent neutropenia  Clinical criteria:  CT with low attenuation lucencies in liver  Mycological criteria:  β-D-glucan >500 pg/mL | ***Candida dubliniensis*** |
| 91 | Proven^4^ | Disseminated | Blood culture positive for *Fusarium solani* | No organisms detected |
| 93 | Proven^5^ | Disseminated | Blood culture positive for *Candida tropicalis* | ***Candida tropicalis*** |
| 106 | Proven  Proven | Pulmonary (*Aspergillus*)  Disseminated (*Rhizopus*) | Lung biopsy culture positive for *Aspergillus fumigatus*  Pathology of skin biopsy with angioinvasive elements, culture positive for *Rhizopus* spp. | HSV-1  *Escherichia coli*  ***Aspergillus fumigatus***  ***Rhizopus oryzae*** |
| 107 | Proven | Disseminated | Pathology of multiple skin biopsies with angioinvasive hyphae, culture positive for *Fusarium* spp. | *Helicobacter pylori*  ***Fusarium vanettenii*** (*Nectria haematococca*) |
| 117 | Proven | Sinus | Pathology with fungal elements and angioinvasion | 1^st^ test: *Staphylococcus epidermidis*  *Enterococcus faecium*  ***Alternaria arborescens***  HHV-7  2^nd^ test: ***Rhizopus oryzae***  HHV-7 15 |
| 119 | Proven^6^ | Pulmonary | Lung biopsy with fungal elements with acute angle branching | No organisms detected |
| 126 | Probable | Sinus | Host criteria: Recent neutropenia  Clinical criteria:  CT with partial sinus opacification  Mycological criteria:  Sinus culture positive for *Alternaria alternata* | No organisms detected |
| 128 | Proven | Disseminated | Blood culture positive for *Candida tropicalis* | No organisms detected |
| 133 | Proven | Sinus | Pathology with fungal elements including septations and acute angle branching; occasional organisms with pigmentation  Tissue culture positive for dematiaceous fungi  (identified as *Exserohilum rostratum* by phenotypic characterization and DNA sequencing) | 1^st^ test: *Eikenella corrodens*  *Abiotrophia defectiva*  ***Aspergillus flavus***  ***Aspergillus oryzae***  CMV  *Enterococcus faecalis*  *Haemophilus parainfluenzae*  *Staphylococcus aureus*  2^nd^ test: *Enterococcus faecalis*  *S. epidermidis*  ***Aspergillus flavus***  ***Aspergillus oryzae*** |
| 135 | Proven | Sinus | Pathology with fungal hyphal elements and angioinvasion, fruiting body-like structures  Culture positive for *Rhizopus* spp. *(*identified as *Rhizopus oryzae* complex by PCR) | ***Rhizopus delemar***  *Enterococcus faecalis* |
| 150 | Proven | Sinus | Pathology with invasive fungal hyphae  Culture positive for *Mucor* spp. | ***Mucor circinelloides***  *Acinetobacter baumannii*  *Staphylococcus epidermidis*  *Corynebacterium matruchotii*  *Actinomyces viscosus*  *Enterococcus gallinarum*  *Veillonella parvula*  *Streptococcus tigurinus*  *Streptococcus mitis*  *Enterococcus faecalis*  *Gardnerella vaginalis*  *Fusobacterium nucleatum*  *Actinomyces oris* |
| 155 | Probable | Pulmonary | Host criteria: Recent neutropenia  Clinical criteria: Chest CT nodule >5 mm  Mycological criteria:  Serum GM 0.810  β-D-glucan 241 pg/mL | ***Aspergillus fumigatus***  *Staphylococcus aureus* (MSSA) |
| 178 | Proven^7^ | Disseminated | Pathology of liver biopsy with yeast forms | ***Candida albicans*** |
| 185 | Proven  Probable | Disseminated  Pulmonary | Blood culture positive for *Candida krusei*  Host criteria: Recent neutropenia  Clinical criteria: Chest CT nodule >5 mm  Mycological criteria:  Serum GM ≥3.750  β-D-glucan >500 pg/mL | ***Candida krusei***  HHV-6B  ***Aspergillus fumigatus*** |
| 193 | Proven^8^ | Sinus | Pathology with fungal elements and angioinvasion | No organisms detected |
| 210 | Proven | Sinus | Pathology with non-septate fungal elements within submucosal tissue  Culture positive for *Rhizopus* spp. *(*identified as *Rhizopus oryzae* complex by PCR) | ***Rhizopus oryzae***  *Rothia mucilaginosa* |
| 216 | Proven | Disseminated | Pathology of skin biopsy with angioinvasive fungal organisms, culture positive for *Fusarium oxysporum* | Adenovirus B |
| 218 | Probable | Sinopulmonary | Host criteria: Recent neutropenia  Clinical criteria:  Chest CT nodule >5 mm  CT with partial sinus opacification  Mycological criteria:  BAL culture positive for *Rhizopus microsporus* | ***Rhizopus microsporus***  CMV  *Escherichia coli*  *Haemophilus parainfluenzae*  *Streptococcus mitis* |
| 219 | Probable | Pulmonary | Host criteria: Recent neutropenia  Clinical criteria:  Chest CT nodule >5 mm, cavitary lesions  Mycological criteria:  Serum GM 0.673 | ***Aspergillus flavus***  ***Aspergillus oryzae***  *Staphylococcus aureus* |
| 232 | Proven  Proven | Disseminated  Pulmonary | Blood culture positive for *Candida parapsilosis*  Pathology of lung biopsy with septated fungal hyphae | ***Candida parapsilosis***  ***Aspergillus niger***  *Lacticaseibacillus rhamnosus* |
| 255 | Proven | Hepatosplenic | Pathology of liver biopsy with fungal organisms | ***Candida albicans*** |
| 263 | Proven^9^ | Pulmonary | Pathology of lung biopsy with fungal organisms | *Staphylococcus saprophyticus*  ***Aspergillus fumigatus*** |
| 275 | Proven^10^ | Pulmonary | Pathology of lung biopsy with fungal hyphae  Culture positive for *Aspergillus fumigatus* | HHV-7  ***Aspergillus fumigatus*** |
| 284 | Proven | Disseminated (skin, lung, GI tract) | Pathology of skin biopsy with fungal hyphal elements  Pathology of colon (after colectomy) with transmural invasive fungal elements  Culture of skin tissue positive for *Aspergillu*s *flavus*  Multiple nodules on chest CT | ***Aspergillus flavus***  ***Aspergillus oryzae***  *Lacticaseibacillus rhamnosus* (*Lactobacillus rhamnosus*)  *Enterobacter cloacae* complex  *Enterococcus faecalis* |
| 285 | Proven | Pulmonary | Pathology of skin biopsy with fungal hyphal elements  Culture positive for *Aspergillus fumigatus* | *Streptococcus agalactiae* |
| 292 | Probable | Pulmonary | Host criteria: Recent neutropenia  Clinical criteria:  Chest CT nodule >5 mm, wedge-shaped consolidation  Mycological criteria:  Serum GM 2.411 | ***Aspergillus fumigatus***  *Enterococcus gallinarum* |

^1^Recent neutropenia: <500 neutrophils/mm^3^ for ≥10 days, temporally related to the onset of fungal disease

^2^The lung biopsy culture for this patient grew *Aspergillus fumigatus* on day 30 of incubation. *Pneumocystis jirovecii* pneumonia was clinically diagnosed clinically and treated earlier in this episode. This patient presented with fever, cough, SOB and hypoxia and had a history of non-adherence to trimethoprim-sulfamethoxazole prophylaxis.

^3^Fungal PCR testing (28S DNA amplification) of biopsied liver tissue was positive for *Candida dubliniensis* DNA.

^4^Skin biopsies also culture positive for *Fusarium solani*.

^5^Skin biopsy showed invasive fungal organisms on pathologic analysis, and fungal PCR testing (28S DNA amplification) of that tissue was positive for *Candida tropicalis* DNA.

^6^Fungal PCR testing (ITS DNA amplification) of biopsied lung tissue was positive for *Aspergillus fumigatus* DNA.

^7^Fungal PCR testing (18S rRNA sequencing) of biopsied liver tissue was positive for *Candida albicans* DNA.

^8^Fungal PCR testing (28S and ITS DNA amplification) of two samples of biopsied sinus tissue were positive for *Alternaria* species DNA, with one sample also positive (ITS DNA amplification) for *Aspergillus fumigatus*.

^9^Fungal PCR testing (ITS DNA amplification) of biopsied lung tissue was positive for *Aspergillus fumigatus* DNA.

^10^Fungal PCR testing (28S and ITS DNA amplification) of biopsied lung tissue was positive for *Aspergillus fumigatus* DNA.

**Supplementary Table S3.** Episodes in which mNGS testing identified a fungus but episode did not meet criteria for proven/probable IFI (false positives)

| **Episode #** | **mNGS result** | **Days from antifungal to mNGS testing** | **Underlying diagnosis** | **Clinical findings** |
| --- | --- | --- | --- | --- |
| 11^1^ | *Lichtheimia ramosa* (formerly *Absidia idahoensis)*  *Agrobacterium tumefaciens*  *Pseudomonas aeruginosa* | 26 | Aplastic anemia s/p SCT  Acute GVH | Parietal scalp cellulitis with abscess  Pathology did not demonstrate invasive disease  18S rRNA sequencing of abscess fluid positive for *Lichtheimia ramosa* |
| 43^1^ | *Candida albicans*  *Prevotella loeschii*  BK polyomavirus  Cytomegalovirus  *Streptococcus intermedius*  *Fusobacterium nucleatum*  *Aspergillus fumigatus*  *Escherichia coli* | -2 | Evan’s syndrome | Presented with acute on chronic PNA, PTX, sepsis  Treated with broad antifungal coverage for presumed disseminated IFI  Continues on secondary prophylaxis with posaconazole  Continues to have chest CT abnormalities, elevated serum BDG |
| 47^1^ | *Bacillus cereus*  *Candida albicans*  *Granulicatella adaciens*  EBV | 6 | Kidney transplant recipient  PTLD | Clinical concern for *Candidal* esophagitis, including by EGD visualization (no bx d/t friable mucosa)  BDG = 163 pg/mL  GM <0.5 from blood and BAL  Abd CT with bowel wall thickening  No 16S/18S testing sent |
| 78 | *Aureobasidium pullulans* | 2 | STAT3 gain-of-function | BDG <31 pg/mL  GM <0.5 from blood and BAL  Chest CT with nodules  BAL cultures no growth  Lung bx cultures no growth  Lung path c/w ILD  GI mucosal path normal  Lung tissue negative on 16S/18S testing |
| 127^1^ | *Candida albicans* | 8 | Burkitt’s lymphoma | ET cultures positive for *C. albicans* at the time of mNGS testing  Liver bx one month later not c/w IFI  Stool and urine positive for *C. albicans* about 2 weeks prior to mNGS testing  History of disseminated *C. albicans* (positive blood cx) four months prior  Imaging c/f esophagitis, typhlitis about 2 weeks prior to mNGS testing  BDG = 294 pg/mL  GM <0.5 from blood  Chest CT with nodules  Abd CT with splenomegaly  Liver bx one month later negative on 16S/18S testing |
| 168^1^ | *Pneumocystis jirovecii*  *Candida albicans* | -2 | ALL | Clinical course consistent with PJP, which was treated  Echinocandin continued for 2 weeks empirically  BDG = 410 pg/mL  GM <0.5 from blood and BAL  Chest CT with nodules  No 16S/18S testing sent |
| 208 | *Candida dubliniensis* | 2 | ALL | Empirically treated with mold-active azole for IPFI  BDG = 31 pg/mL  GM <0.5 from blood and BAL  Chest CT with nodules and cavitary lesions  Abd CT without abnormalities  No 16S/18S testing sent  *Candida* not considered likely cause of illness per ID notes |
| 243 | *Candida albicans*  *Enterococcus faecalis* | 3 | Recurrent ectomesenchymoma | *Candida* not considered likely cause of illness per ID notes |

^1^Episode in which the results of standard microbiologic testing and the clinical impression were consistent with the organism identified by mNGS testing

**Supplementary Table S4.** Episodes which met criteria for proven/probable IFI in which mNGS testing did not identify a fungus (false negatives)

| **Episode #** | **IFI diagnosis** | **mNGS result** | **Days from antifungal to mNGS testing** | **Underlying diagnosis** | **Clinical findings** |
| --- | --- | --- | --- | --- | --- |
| 72 | Proven pulmonary *Aspergillus fumigatus* | *Pneumocystis jirovecii*  Torque Tenovirus | 1 | ALL | Febrile neutropenia, hypoxemia  Chest CT with nodular opacities  *Aspergillus* growth from lung tissue on day 30 of incubation |
| 91 | Proven disseminated *Fusarium solani* | No organisms identified | 26 | ALL | Febrile neutropenia  Diffuse skin rash  *Fusarium solani* growth from blood and skin biopsy samples |
| 119 | Proven pulmonary *Aspergillus fumigatus* | No organisms identified | 4 | ALL | Febrile neutropenia  Chest CT with nodular opacities  *Aspergillus fumigatus* DNA amplified from lung biopsy tissue |
| 126 | Probable sinus *Alternaria alternata* | No organisms identified | 2 | ALL | Febrile neutropenia  Sinus CT with partial opacification and bony changes |
| 128 | Proven disseminated *Candida tropicalis* | No organisms identified | 6 | ALL | Febrile neutropenia  Blood cultures cleared after one day of treatment |
| 193 | Proven sinus *Alternaria* and *Aspergillus fumigatus* | No organisms identified | 0 | AML | Febrile neutropenia  Sinus opacification on CT scan  *Alternaria* spp. and *Aspergillus fumigatus* DNA amplified from sinus biopsy tissue |
| 216 | Proven disseminated *Fusarium oxysporum* | Adenovirus B | 26 | ALL | Febrile neutropenia  Polymicrobial bacteremia (*Escherichia coli*, *Rothia mucilaginosa*) with sepsis  *Fusarium oxysporum* cultured from skin biopsy tissue |
| 285 | Proven pulmonary *Aspergillus fumigatus* | *Streptococcus agalactiae* | 1 | ALL | Febrile neutropenia  Polymicrobial bacteremia with sepsis  Disseminated *Streptococcus agalactiae* (positive blood and skin biopsy cultures)  *Aspergillus fumigatus* and *Enterococcus faecalis* cultured from lung biopsy tissue |
